# *Escherichia coli* from six European countries reveals differences in profile and distribution of critical antimicrobial resistance determinants within One Health compartments, 2013 to 2020

**DOI:** 10.2807/1560-7917.ES.2024.29.47.2400295

**Published:** 2024-11-21

**Authors:** Håkon P Kaspersen, Michael SM Brouwer, Javier Nunez-Garcia, Ingrid Cárdenas-Rey, Manal AbuOun, Nicholas Duggett, Nicholas Ellaby, Jose Delgado-Blas, Jens A Hammerl, Maria Getino, Carlos Serna, Thierry Naas, Kees T Veldman, Alex Bossers, Marianne Sunde, Solveig S Mo, Silje B Jørgensen, Matthew Ellington, Bruno Gonzalez-Zorn, Roberto La Ragione, Philippe Glaser, Muna F Anjum

**Affiliations:** 1 Norwegian Veterinary Institute, Section for Food Safety and Animal Health Research, Ås, Norway; 2 Wageningen Bioveterinary Research part of Wageningen University and Research, Department of Bacteriology, Host-Pathogen interactions and Diagnostic Development, Lelystad, The Netherlands; 3 Department of Bacteriology, Animal and Plant Health Agency, Addlestone, United Kingdom; 4 United Kingdom Health Security Agency, London, United Kingdom; 5 Antimicrobial Resistance Unit, Animal Health Department, Faculty of Veterinary Medicine and VISAVET Health Surveillance Centre, Complutense University of Madrid, Madrid, Spain; 6 Department of Biological Safety, German Federal Institute for Risk Assessment, Berlin, Germany; 7 School of Veterinary Medicine, Faculty of Health and Medical Sciences, University of Surrey, Guildford, United Kingdom; 8 Bacteriology-Hygiene unit, Hopital Bicêtre, Assistance Publique-Hopitaux De Paris, University Paris-Saclay, Paris, France; 9 Department for Microbiology and Infection Control, Department for Emergency Medicine, Akershus University Hospital, Lørenskog, Norway; 10 School of Biosciences, Faculty of Health and Medical Sciences, University of Surrey, Guildford, United Kingdom; 11 Ecology and evolution of antibiotic resistance Unit, Institut Pasteur, Université Paris Cité, Paris, France

**Keywords:** Antimicrobial resistance, whole genome sequencing, One Health, surveillance

## Abstract

**Background:**

Antimicrobial resistance (AMR) is a global threat. Monitoring using an integrated One Health approach is essential to detect changes in AMR occurrence.

**Aim:**

We aimed to detect AMR genes in pathogenic and commensal *Escherichia coli* collected 2013–2020 within monitoring programmes and research from food animals, food (fresh retail raw meat) and humans in six European countries, to compare vertical and horizontal transmission.

**Methods:**

We whole genome sequenced (WGS) 3,745 *E. coli* isolates**,** detected AMR genes using ResFinder and performed phylogenetic analysis to determine isolate relatedness and transmission. A BLASTn-based bioinformatic method compared draft IncI1 genomes to conserved plasmid references from Europe.

**Results:**

Resistance genes to medically important antimicrobials (MIA) such as extended-spectrum cephalosporins (ESC) were widespread but predicted resistance to MIAs authorised for human use (carbapenem, tigecycline) was detected only in two human and three cattle isolates. Phylogenetic analysis clustered *E. coli* according to phylogroups; commensal animal isolates showed greater diversity than those from human patients. Only 18 vertical animal-food and human-animal transmission events of *E. coli* clones were detected. However, IncI1 plasmids from different sources and/or countries carrying resistance to ESCs were conserved and widely distributed, although these variants were rarely detected in human pathogens.

**Conclusion:**

Using WGS we demonstrated AMR is driven vertically and horizontally. Human clinical isolates were more closely related, but their IncI1 plasmids were more diverse, while animal or food isolates were less similar with more conserved IncI1 plasmids. These differences likely arose from variations in selective pressure, influencing AMR evolution and transmission.

**Key public health message**

**What did you want to address in this study and why?**

Antimicrobials are crucial for treatment of bacterial infections. The presence of resistant bacteria weakens our ability to treat disease. We wanted to investigate the types of resistance that may be present in *Escherichia coli* that can be harmless, but also cause a range of gastrointestinal and urinary tract or blood infections, that were isolated from human patients, food animals and fresh retail raw meat from six European countries.

**What have we learnt from this study?**

Resistance to antimicrobials essential for treating human infections was widespread across Europe and all sample types, while resistance was relatively low to antimicrobials highly critical for treatment. *Escherichia coli* more frequently carried the resistance genes on DNA fragments or plasmids which moved between different *E. coli*. The plasmids were less related to each other in human isolates than those from other sample types.

**What are the implications of your findings for public health?**

Humans, food and livestock often carry resistant bacteria. While resistance is most relevant in bacteria causing infections, similarly, commensal bacteria may become resistant. Therefore, we need to understand in more detail which factors contribute to the spread of resistance among both pathogenic and commensal *E. coli.*

## Introduction

The rising global threat of antimicrobial resistance (AMR) in bacteria, including resistance to medically important antimicrobials (MIAs) reserved primarily or exclusively for the treatment of human infections [1], has been termed as the silent pandemic as it is a challenge and a critical health problem [2]. Antimicrobial resistance is considered a One Health issue due to the interconnectedness of compartments such as humans, animals and food. However, the level of bacterial transmission and attribution of each compartment to the other is still open for discussion, as results from studies are variable [3,4].

Within the European Union (EU) and the United Kingdom (UK), harmonised annual AMR monitoring programmes in healthy livestock and food provide insight into the occurrence of resistance to different antimicrobials, including MIAs, in zoonotic and commensal bacterial species [5]. These programmes include monitoring *Escherichia coli* harbouring resistance to extended-spectrum (second or third) cephalosporins (ESCs), such as AmpC beta-lactamases or extended-spectrum beta-lactamases (ESBL), which can cause opportunistic infections, complicating disease treatment. Increased occurrence of AMR is thought to largely be due to overuse or misuse of antimicrobials in different sectors [6]. In Europe, a concerted effort to reduce antimicrobial usage, particularly in livestock [7], and a ban on the use of antimicrobials for growth promotion [8] has reduced the occurrence of resistant bacteria in healthy food animals entering the food chain [9]. Due to the lack of continuous monitoring of commensal *E. coli* in healthy humans, it is difficult to estimate AMR trends in the community. However, available data suggest *E. coli* from samples from invasive infections in humans (e.g. cerebrospinal fluids, blood, etc), with resistance to MIAs, only slightly reduced between 2017 and 2021 [10].

Whole genome sequencing (WGS) of bacterial isolates is a useful tool for outbreak investigations and transmission analysis, for detection of AMR mechanisms and monitoring trends [11-13]. The technology has developed rapidly over the past decade, allowing the detection of genes encoding resistance to antimicrobials and virulence determinants; there is currently a plethora of methods available for their detection [14]. Phylogenetic analysis can identify the evolutionary origin of AMR-harbouring isolates, allowing comparison of their genomes [15,16]. In fact, due to the increase in availability of relevant genome sequences, comparative analyses of human clinical isolates with isolates from healthy livestock have now become possible, so the relative contribution of these compartments to AMR proliferation can be better assessed [17].

Transmission of AMR can be vertical through replication of AMR-harbouring bacteria or horizontal, with mobile genetic elements such as plasmids transferring AMR genes between unrelated bacterial strains or species. Some WGS methods allow tracking of AMR through reconstruction of genomes with higher accuracy. However, the plasticity of plasmid genomes, with segments of DNA often being shared between a plasmid and its host, makes it problematic to reconstruct them from using short-read data produced from Illumina (Illumina, San Diego, the United States (US)) sequencing technology [18]. Therefore, unless AMR genes are assembled into the same contiguous genomic fragment (contig), which also contains the plasmid replicon or core chromosomal genes [19], it is impossible to discern whether AMR genes belong to a plasmid and have been horizontally transferred or vertically through bacterial replication. Long-read sequencing has helped solve this problem; however, it remains too expensive to use at the scale required for surveillance.

Within the One Health European Joint Programme (OH-EJP) Antibiotic Resistance Dynamics (ARDIG) project [20], we used a One Health approach to explore the impact of antimicrobials on humans, animals and food (fresh retail raw meat), across six European countries. The aim of this study was to compare AMR in *E. coli* isolates by analysing AMR genes, phylogeny and AMR plasmid distribution across participant countries using short-read WGS data to assess transmission between the food chain and humans. We used published bioinformatic tools to detect AMR genes and phylogenetic distribution of isolates carrying AMR and developed a novel approach based on Basic Local Alignment Search Tool (BLAST) to identify likely AMR plasmid transmission. As a proof-of-principle, we focused on IncI1 replicon-type plasmids, one of the most abundant AMR harbouring plasmids and a reference set of circularised IncI1 plasmid genomes.

## Methods

### Isolate collections

A total of 3,745 *E. coli* isolates originated from samples (i) from several different longitudinal studies with convenience sampling (n = 2,123) [20-24] and (ii) collected from national harmonised monitoring of AMR in livestock and retail meat in Europe (n = 1,622) [25]. All animals sampled within the national monitoring programmes were domestically raised. Animal samples from national monitoring programmes and longitudinal studies were from faeces of healthy livestock.

In longitudinal studies from human patients, 1,058 *E. coli* isolates were from clinical specimens of urinary tract infections, faecal samples or blood stream infections analysed at diagnostic laboratories. These isolates were collected monthly over 1 year from each participating country. The travel history of the patients was unknown.

Longitudinal studies on poultry were carried out on 27 farms in Norway, one farm in the Netherlands and one in the UK. Broilers were sent to slaughter every 4–6 weeks and there was ca one sampling per month performed over 9–12 months. Two UK pig farms were sampled longitudinally where pigs were sent to slaughter ca every 3 months, there were 3–12 samplings performed over 12–18 months. For cattle, the Dutch *E. coli* isolates were from 683 cattle born on 13 different dairy farms and subsequently sent to eight different veal farms, where they were a subset of a much larger population; sampling was at five time points over 6 months [21]. The UK cattle isolates were from a mixed farm comprising 100 cows which were sampled monthly.

Food samples, which included imported food, were from fresh retail raw meat collected from major supermarkets. See Table 1 for isolate details which includes numbers from each compartment (humans: n = 1,058; livestock longitudinal: n = 1,065; livestock national monitoring: n = 1,308; retail meat: n = 314), country (France: n = 243; Germany: n = 118; the Netherlands: n = 585; Norway: n = 280; Spain: n = 378; UK: n = 2,141), year of isolation (2013: n = 173; 2015: n = 242; 2016: n = 662; 2017: n = 535; 2018: n = 580; 2019: n = 1,423; 2020: n = 130) and whether isolated using antibiotic selective (n = 1,932) or non-selective media (n = 1,813). The authors make available the full isolate list, source, sampling type and isolation method in Supplementary Table S1.

**Table 1:** *Escherichia coli* isolates collected from France (n = 243), Germany (n = 118), the Netherlands (n = 585), Norway (n = 280), Spain (n = 378) and the United Kingdom (n = 2,141), 2013–2020

Sampling and sample type	Isolates n = 3,745	Culture media		Year	Country
		Selective^a^ n =1,932	Non-selective n = 1,813		
Longitudinal sampling					
Human samples (n = 1,058)					
UTI	936	0	936	2017, 2019–2020	ES, FR, NO, UK
Blood	107	0	107	2017–2018	UK
Faeces	15	0	15		
Livestock (n = 1,065)					
Pig^a^	481	188	293	2018–2019	UK
Cattle	334	320	14	2018–2020	NL, UK
Poultry	250	99	151	2016–2019	NL, NO, UK
National monitoring					
Livestock (n = 1,308)					
Pig	734	587	147	2013–2019	DE, ES, NL, UK
Cattle	125	121	4	2016–2017	DE, NL
Poultry	449	308	141	2015–2018	DE, ES, NL, UK
Retail meat (n = 314)					
Pork	19	14	5	2015–2019	DE, UK
Beef	1	1	0	2017	DE
Poultry meat	294	294	0	2016–2018	UK

DE: Germany; ES: Spain; FR: France; NL: the Netherlands; NO: Norway; UK: United Kingdom; UTI: urinary tract infection.

^a^ *Escherichia coli* selective plates included selection of cephalosporin-resistant *E. coli* for all studies except one UK pig study which included selection of cephalosporin- and ciprofloxacin-resistant *E. coli* [22].

### Bioinformatic analyses

Illumina Nextera libraries were prepared from extracted *E. coli* DNA which were sequenced using Illumina MiSeq, HiSeq or NextSeq. The methods used for DNA extraction and sequencing are given in Supplementary Table S2. Sequencing reads were trimmed, assembled and quality controlled, and genome sequences were deposited at the European Nucleotide Archive (https://www.ebi.ac.uk/ena/browser/home) under study accession number PRJEB64161. Assembled genomes were analysed for AMR genotypes using ResFinder [26] (version 4.0) and PlasmidFinder [27] (version 2.0.1) to detect plasmid replicon types, including identifying isolates with IncI1. In silico multilocus sequence typing (MLST) was performed using mlst [28] (version 2.16.4). A core gene alignment was generated with Panaroo [29] (version 1.2.7) for all genomes, and a phylogenetic tree was reconstructed from the alignment with IQ-Tree [30] (version 1.6.12). Putative transmission clusters (PTCs) were defined by clustering the core gene single nucleotide polymorphism (SNP) distances by single linkage, followed by cutting the resulting tree with the cutree function in Rdocumentation (https://www.rdocumentation.org/packages/stats/versions/3.6.2/topics/cutree) using a SNP distance of 50. Each cluster was assigned an arbitrary numerical identification code (ID). The PTCs with at least five isolates and containing isolates from more than one country were separately analysed with the core genome track of ALPPACA (version 2.2.0) pipeline [31], to generate phylogenies with a higher resolution. Transmission events were defined as all instances where there was a core genome SNP distance of < 50 between any two isolates from different countries from ALPPACA.

### IncI1 plasmid identification

A reference panel of 181 circularised IncI1 plasmids from European isolates was assembled for identification of conserved plasmids present in our short-read assemblies. These plasmids represented seven European countries (France, Germany, Italy, the Netherlands, Norway, Switzerland, UK), different years (2005–2020), plasmid length (18.5–207 kbp), sources (cattle, dogs, humans, mollusc, pig, pork, poultry, sheep, water, unknown) and AMR content (0–7 AMR genes). The IncI1 plasmids included were: (i) 30 previously described in *E. coli* [32], (ii) 105 from The National Center for Biotechnology Information (NCBI) plasmid database of which 81 were present in *E. coli*, 10 in *Escherichia fergusonii*, five in *Shigella sonnei*, four in *Salmonella enterica*, two in *Escherichia marmotae*, one in *Shigella flexneri*, one in *Citrobacter freundii* and one in *Enterobacter hormaechei* and (iii) 46 from REHAB project [33], all from *E. coli* with both long- and short-read WGS data available. A dendrogram for the reference plasmids was generated using MashTree [34] (version 1.2.0). The authors have made available additional analyses comparing the reference plasmids in Supplementary Figure S1.

The 1,194 assemblies produced from WGS data from ARDIG isolates containing an IncI1 replicon were subsequently compared by finding regions of nucleotide sequence similarity using BLASTn against the reference plasmid genomes. Contigs with ≥ 98% coverage and ≥ 99% identity to any reference plasmid genome were used to build up an accumulative coverage function over the reference set. The authors have made available an analysis of the percentage coverage in reconstructed IncI1 plasmids, with respect to the reference panel in Supplementary Figure S2. Each isolate was assigned to the plasmid in the reference panel with highest percentage of genome matched which has been made available in Supplementary Table 3. These strict thresholds were set to be certain that the IncI1 (AMR carrying) plasmids present in our isolates indeed matched the reference plasmid or was highly like it. The method was validated using a set of isolates for which long and short-read WGS was already available, as described in Supplementary Materials and Methods.

## Results

### Detection of antimicrobial resistance genes

**Figure 1:**
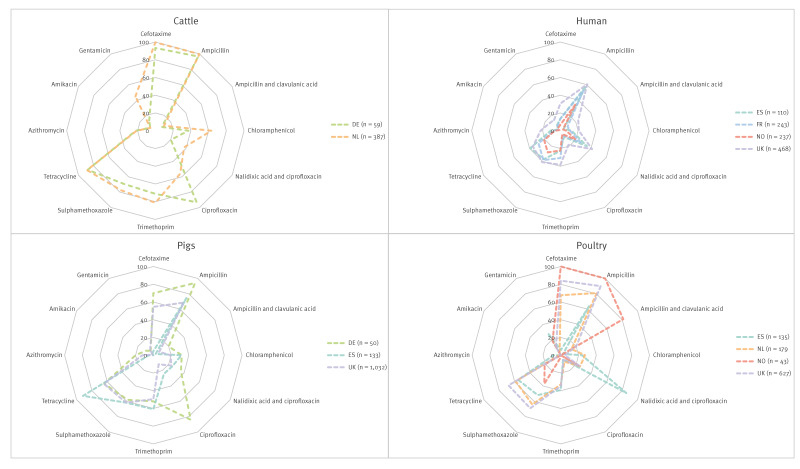
Percentages of predicted genotypic resistance to different antimicrobial classes in *Escherichia coli* isolates, by isolate source, France, Germany, the Netherlands, Norway, Spain, the United Kingdom, 2013–2020 (n = 3,745)

DE: Germany; ES: Spain; FR: France; NL: the Netherlands; NO: Norway; UK: the United Kingdom.

The resistance genotypes to all antimicrobials present in the EU AMR monitoring panel for *E. coli* from all compartments are presented in Figure 1. It shows that human isolates, cultured from non-selective media, overall had lower occurrence of AMR genes than other compartments where *E. coli* were cultured from a mixture of antibiotic selective and non-selective media. Despite this, human *E. coli* harboured a variety of AMR genes, as the authors demonstrate in Supplementary Table S4, enabling comparison to *E. coli* from retail meat and livestock. Our analysis focused on (i) beta-lactam and fluoroquinolone resistance genes and mutations, as these are MIAs for disease treatment and (ii) tetracycline, the most prevalent AMR class to which resistance was detected. The authors provide an additional list of the actual numbers of isolates harbouring AMR genes within each AMR class presented in Figure 1, for each country and compartment, in Supplementary Table S4.

Numbers in the central Y-axis of each spider graph indicate the proportions of resistance for each antimicrobial. All proportions based on fewer than 30 isolates (14 UK cattle isolates, 19 NL pig isolates and 9 DE poultry isolates) have been removed.

**Figure 2:**
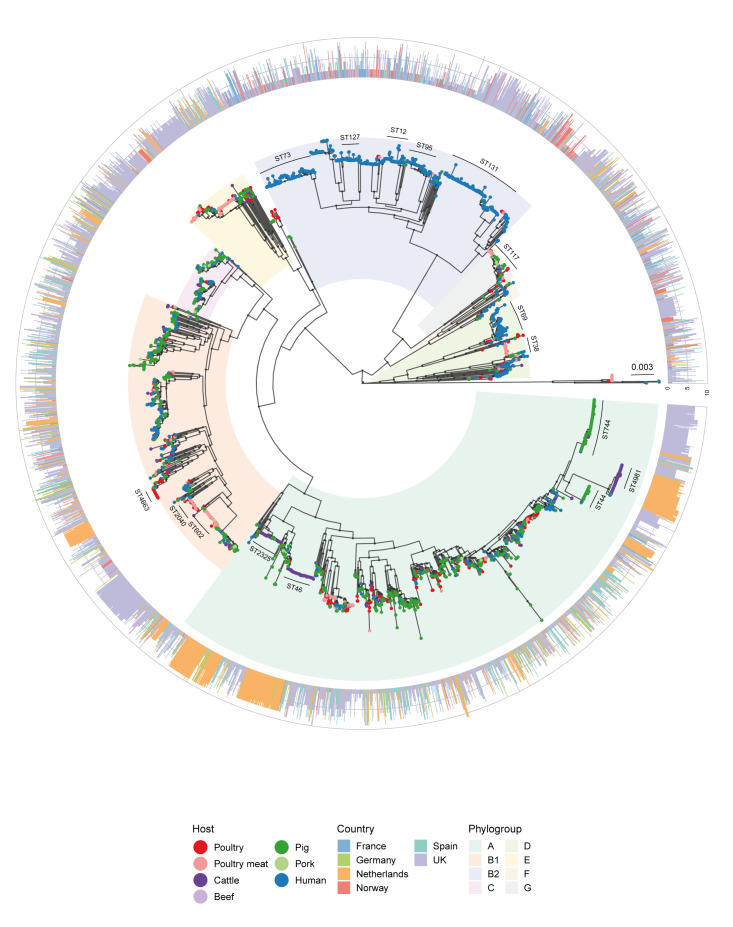
Maximum likelihood phylogeny based on an alignment of 3,103 core genes from *Escherichia coli* isolates from France, Germany, the Netherlands, Norway, Spain, the United Kingdom, 2013–2020 (n = 3,745)

Cefotaxime (an ESC) resistance was widely predicted in the isolates (n = 1,975; 52.7%), as presented in Supplementary Table S4. Extended-spectrum beta-lactamases, particularly *bla*_CTX-M-1_, were present in most animal/country combinations, but there was a much greater diversity of ESBL genes in human isolates. The *bla*_SHV-12_ was more abundant in isolates from German and Spanish broilers, while various types of *bla*_TEM_ were present in isolates from Spanish pigs, including several ESBL variants. Resistance to the ampicillin-clavulanic acid combination, deduced from presence of plasmid-encoded AmpC, were most prominent in isolates from Norwegian poultry in which *bla*_CMY-2_ was the most abundant gene. Two chromosomal *ampC*-promotor mutations (-42 C > T and -32 T > A) predicted to cause AmpC hyperproduction were detected. The -42 C > T mutation was detected in isolates from veal and cattle in the Netherlands, pigs in Germany and pigs, pork, broilers, chicken meat and turkey in the UK. The -32 T > A mutation was detected exclusively in isolates from humans from Spain and the UK. Ampicillin resistance was common (2,161/2,687, 80.4%) in isolates from all countries in livestock and meat, while less common in human collections (554/1,058, 52.4%; Figure 2) which may be due to differences in the sample collection and isolation methods, Supplementary Table S1. Carbapenemases were only detected in two human isolates, one from the UK harbouring *bla*_NDM-1_ and one from France harbouring *bla*_OXA-181_; neither were included in Figure 1.

UK: United Kingdom.

Shaded areas with colour represent the major phylogroup of that clade. Nodes with black dots represent accepted bootstrap values (≥ 95). Tip point colours represent host species. Major sequence types of interest are marked with a line for each clade. The bar plot on the rim counts the number of antimicrobial types with at least one antimicrobial resistance gene present for each sample, and the colour represents the country of origin. Evolutionary model: GTR + F + R6. The tree is midpoint rooted [43].

Predicted reduced susceptibility to ciprofloxacin and/or nalidixic acid through acquired resistance genes or chromosomal mutations were detected. Acquired resistance mechanisms were relatively rare in human isolates in Norway, Spain and France (9/237, 10/110, 17/243, respectively; 3.8–9.1%), and were mostly due to presence of *qnrS1*. However, occurrence was higher in the UK human isolates (89/468; 19.0%), primarily due to a higher frequency of aac(6’)-Ib-cr, (Figure 1), which also causes resistance to aminoglycosides. In pig isolates, resistance varied by country, with none of the 19 isolates in the Netherlands, 11.8% in the UK (122/1,032), 24.1% in Spain (32/133), and 84% (42/50) in pig and pork isolates from Germany, harbouring acquired ciprofloxacin resistance. In poultry isolates, it was only 5.2–8.4% in Spain (7/135), the Netherlands (15/179), the UK (39/627, including poultry meat), while all nine German isolates were resistant. None of the 43 Norwegian isolates was resistant. In cattle, none of the 14 UK isolates were resistant but 56.1% in the Netherlands (217/387) and 55 of 59 German cattle and beef isolates were resistant. Resistance to ciprofloxacin in animal and meat isolates was due to *qnrS1*, although a notable proportion of poultry isolates from Germany and Spain also harboured *qnrB1* and *qnrB19*.

Resistance to ciprofloxacin and nalidixic acid due to chromosomal point mutations in *gyrA*, often in combination with *parC* and *parE*, differed in comparison to plasmid-encoded resistances. In human isolates, resistance to quinolones due to chromosomal mutations was higher in all countries than plasmid-acquired resistance genes. The former ranged between 19.4% (Norway 46/237) and 41.2% (UK 193/468). It was ca 25% in poultry isolates from the Netherlands (45/179) and the UK (155/627, including poultry meat isolates), while it was 85.2% in Spain (115/135). Of the German isolates, 2 of 9 were resistant, as were 10 of the 43 Norwegian ones. The occurrence of chromosomal mutations was notably lower (18/50) than acquired resistance genes in pigs and pork from Germany. It was ca 21-22% in Spain (28/133) and the UK (225/1,032), with chromosomal mutations being more common than acquired resistance genes in the latter. Only one of the 19 Dutch isolates harboured chromosomal mutation. Finally, resistance in cattle isolates ranged from no resistance among the 14 isolates in the UK to 12 of 59 isolates in Germany and 37.5% in the Netherlands (145/387). While mutations in *gyrA* and *parC* were detected in all countries and species, mutations in *parE* were mainly detected in human and cattle isolates in all countries.

Tetracycline resistance was predicted in isolates from all sample types. In humans, the occurrence of resistance genes ranged from 21.1% (Norway: 50/237) to 39.1% (Spain: 43/110) (Figure 1). In poultry isolates, it was more diverse, with 9 of 43 Norwegian and 4 of 9 German isolates, 55.5% in Spain (75/135), 58.7% in the Netherlands (105/179) and 67.9% in the UK (426/627, including poultry meat). In pig isolates, the frequency varied, ranging from 9 of 19 isolates in the Netherlands, 32 of 50 isolates in Germany (including pork), 64.3% in the UK (664/1,032, including pork) to 91.7% in Spain (122/133). The overall occurrence was the highest in cattle and beef isolates from Germany (52/59) and the Netherlands at 88. 9% (344/387), while no tetracycline resistance genes were detected in the low number (n = 14) of UK cattle isolates. The *tet*(A) gene was most frequent in all countries and species, while *tet*(B) and *tet*(M) were also detected in some. The *tet*(X6) gene, which confers resistance to tetracycline and tigecycline, a last resort antibiotic in humans, was found in three cattle isolates from the Netherlands.

### Pangenome analysis and phylogenetic reconstruction

The pangenome analysis detected 41,867 genes, of which 3,103 were regarded as core genes. Among the 3,745 isolates included, 543 unique sequence types (ST) were detected (Simpson diversity 0.981), where 282 STs (51.9%) were only represented by a single isolate, indicating isolates were highly diverse.

The phylogenetic analysis revealed several major clusters according to phylogroups (Figure 2). There were 181 isolates of which two were not assigned a group and 179 did not cluster with the rest of the isolates from the same phylogroup. For the ease of interpretation, each phylogroup represented by the highest number of isolates within a clade in the phylogenetic tree was referred to as the major phylogroup in that clade. As expected, most (687/1,058) of human isolates were within phylogroup B2, which also harboured a handful (n = 46) of isolates from livestock or livestock products. Human isolates were the largest source in phylogroups D (138/284, 48.6%) and F (38/66, 57.6%) as well, with handfuls present in other phylogroups. Isolates within phylogroup B2, D and F originated mostly from samples from urinary tract infections (n = 595, n = 128 and n = 34, respectively). Livestock and food isolates were genetically more diverse as indicated from phylogroups, and were present in all phylogroups, although generally less in B2, D and F.

### Vertical transmission clusters

A total of 75 PTCs were identified among all isolates from different species and/or countries as authors have presented in Supplementary Table S5. Of these, 15 were of further interest as the isolates were distributed between different countries or host species (Table 2). All phylogroups, except phylogroup E, were represented. A total of 14 different STs were represented among the 15 PTCs. Three clusters contained only human isolates from different countries, all from phylogroup B2. Three clusters contained isolates identified to harbour IncI1 AMR plasmids. Of these, two clusters comprised poultry and poultry meat isolates from the Netherlands, Norway and the UK and one comprised pig and pork isolates from Spain and the UK.

**Table 2:** Overview of putative transmission clusters of *Escherichia coli* isolates from France, Germany, the Netherlands, Norway, Spain, the United Kingdom, 2013–2020 (n = 3,745)

Cluster	Phylogroup	ST	Sampling strategy		Source	Country	Plasmid	Average genome coverage (%)	SNP distance		
			Monitoring	Longitudinal					Mean	Median	Range
44^a^	A	46	1	56	Cattle	DE, NL	ND	89.50	16	15	1–39
23^b^	A	2040	29	5	Poultry, poultry meat	NO, UK	IncI (n = 3)	86.80	32	30	2–91
449^a^	G	117	17	0	Poultry, poultry meat	NL, UK	IncI (n = 6)	93.30	43	46	1–73
193^b^	B2	429	7	6	Human, poultry, poultry meat	FR, NL, NO, UK	ND	86.80	28	20	4–88
174^a^	A	46	1	11	Cattle	DE, NL	ND	86.40	22	22	2–43
419^a^	A	2325	2	9	Cattle	DE, NL	ND	89.50	22	16	8–41
51^a^	A	744	2	9	Cattle, pig, poultry	ES, NL, UK	ND	92.40	32	29	1–68
580	B2	1193	2	7	Human	ES, FR, NO, UK	ND	86.50	78	80	50–114
509	B2	404	1	8	Human	ES, FR, NO, UK	ND	80.30	86	87	63–101
64	C	410	6	0	Pig, pork	ES, UK	IncI (n = 3)	87.30	82	88	25–122
764^a^	D	69	2	5	Human, pig, poultry	ES, FR, UK	ND	87.20	74	75	50–94
1185^b^	B1	683	6	0	Cattle, pig	DE, NL, UK	ND	90.50	22	24	7–28
1123	B2	131	1	4	Human	ES, NO, UK	ND	88.00	67	66.5	53–80
346	B1	58	3	2	Human, pig, poultry, poultry meat	NO, UK	ND	90.80	56	59.5	10–69
417^a^	F	1722	2	3	Cattle, human	DE, NL, NO	ND	93.60	54	49.5	17–89

DE: Germany; ES: Spain; FR: France; ND: not detected; NL: the Netherlands; NO: Norway; PCT: putative transmission cluster; SNP: single nucleotide polymorphism; ST: sequence type; UK: the United Kingdom.

^a^ PCTs which were deduced as vertical transmission events from core genome SNP distances; for ST1722 only cattle isolates were inferred.

^b^ PCTs which were associated with more than one vertical transmission event.

**Figure 3:**
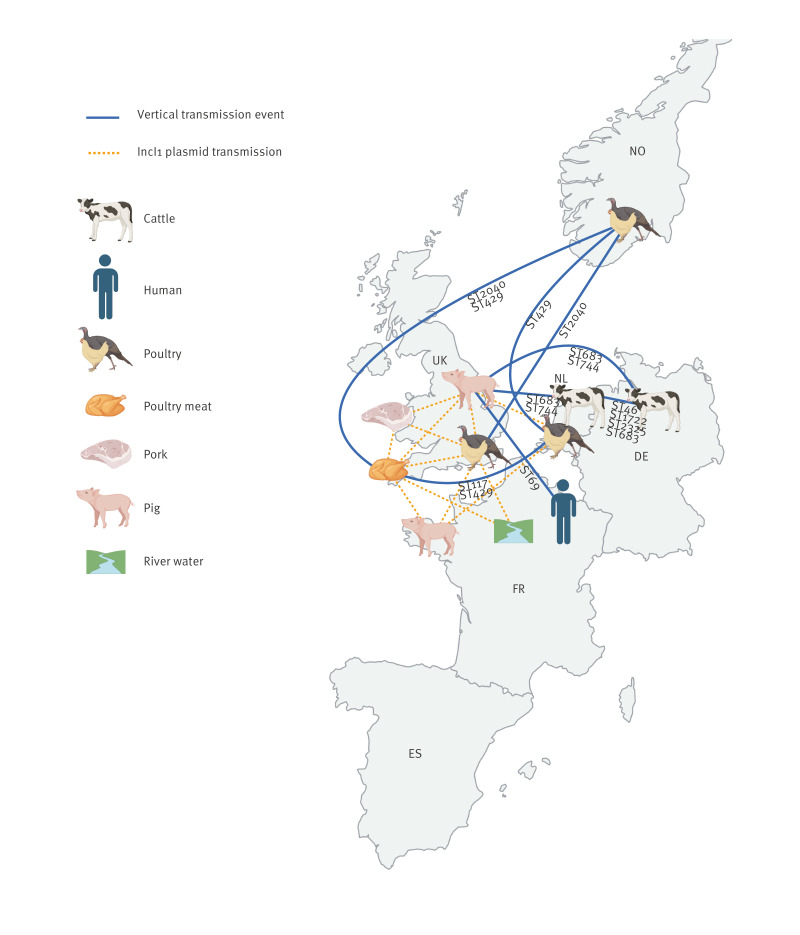
Transmission events for *Escherichia coli* isolates and conserved IncI1-plasmids in different sample types from France, Germany, the Netherlands, Norway, Spain, and the United Kingdom, 2013–2020 (n = 3,745)

DE: Germany; ES: Spain; FR: France; NL: the Netherlands; NO: Norway; SNP: single nucleotide polymorphism; ST: sequence type; UK: the United Kingdom.

Vertical transmission events (TEs) were inferred from the core genome SNP distances in ten of the 15 PTCs (Table 2). Overall, 18 TEs were detected, with more than one TE associated with three PCTs (Table 2). The authors have provided in Supplementary Table S6 additional data regarding the host species, countries and STs for isolates for each TE. The TEs harboured isolates from nine different STs: ST2040, ST117 and ST429 were associated with poultry and poultry meat; ST46, ST2325, and ST1722 were associated with cattle, while ST683, ST744 and ST69 were associated with more than one host (Figure 3). Transmission events with the highest number of isolates involved were *E. coli* ST46 (n = 57) from cluster 44 comprising cattle isolates from the Netherlands and Germany (mean SNP distance = 21.4; from 2017, 2019 and 2020), followed by ST2040 (n = 24) from cluster 23, including poultry isolates from Norway and the UK (mean SNP distance = 29.7; from 2016 and 2018). Interestingly, only one TE with human isolates was detected, it involved a human isolate from France and a pig isolate from the UK, both ST69 (mean SNP distance = 50), but separated by 4 years (2015–2019). Only one group of TEs within cluster 23 harboured IncI1 plasmids. These were all *E. coli* from poultry meat in the UK. It included two isolates carrying the IncI1 ESBL318-reference-like plasmid with *bla*_CMY-2_; three isolates carrying the IncI1 LREC4925-reference-like plasmid with *bla*_CTX-M-1_, *sul2*, *tetA*; and one isolate carrying the IncI1 NZ_MT230105.1-reference-like plasmid harbouring *bla*_CTX-M-1_, *sul2*, *tetA*; the reference plasmid details are given in Supplementary Table S3. The other closely related isolates within the same TE did not harbour any IncI1 plasmids. No directionality could be inferred in these vertical transmissions except from poultry-to-poultry meat.

Vertical transmission events are represented by blue lines, and IncI1 plasmid transmission is shown with dotted orange lines. All vertical transmission events are based on a core genome SNP distance of ≤ 50, only across countries. Sequence types within each vertical transmission event are noted below each blue line and IncI1 plasmids are in yellow. The location of each host species/sample material within each country is arbitrary. The figure was created with BioRender (https://www.biorender.com/).

### IncI1 plasmid spread and AMR profiles

Twenty-three Inc groups were detected in 88.1% (n = 3,299) of isolates with several containing multiple Inc groups. The most common were IncF, present in 77.0% (n = 2,883) of all isolates, then IncI (n = 1,268; 33.85%) and IncX (n = 576; 15.38%). The authors have compared the distribution of different plasmid Inc types in Supplementary Figure S3. However, both IncF and IncI comprise multiple subtypes, with IncFIA, IncFIB, IncFIC, IncFII and IncI1, IncI2 and IncIγ for the IncF and IncI groups, respectively. From these subtypes, IncI1 was detected in 1,194 isolates (31.9%), representing the largest group, and was therefore the focus of this study. Within the IncI1 harbouring subgroup, 429 isolates were from pigs, 337 from poultry, 244 from poultry meat, 117 from humans, 56 from cattle and 11 from pork. From the PlasmidFinder data (Table 3), we noted IncI1 plasmids were present in a lower proportion of isolates from Spain, Norway and France (18.8%, 15.7% and 14.0%, respectively) than Germany and the Netherlands (25.4% and 28.0%) and the UK (39.7%).

**Table 3:** *Escherichia coli* isolates from France, Germany, the Netherlands, Norway, Spain, the United Kingdom harbouring a conserved IncI1 reference plasmid, 2013–2020 (n = 3,745)

Country	Number of isolates	IncI1 plasmids in reference panel	Isolates with an IncI1 replicon identified from ResFinder		Percentage of isolates with an IncI1 replicon n = 1,194	Isolates with an IncI1 plasmid with > 98% match with reference panel		Percentage of isolates with an IncI1 plasmid with > 98% match in reference panel n = 410
			n	%		n	%	
DE	118	7	30	25.4	2.5	4	3.4	1.0
ES	378	0	71	18.8	5.7	3	0.8	0.7
FR	243	17	34	14.0	2.8	2	0.8	0.5
NL	585	27	164	28.0	13.7	36	6.2	8.8
NO	280	2	44	15.7	3.7	0	0.0	0.0
UK	2,141	112	851	39.7	71.3	365	17.0	89.0
Total	3,745	183^a^	1,194	31.9	99.7	410	10.9	100

DE: Germany; ES: Spain; FR: France; NL: the Netherlands; NO: Norway; UK: the United Kingdom.

^a^ Additional countries: Italy (n = 4) and Switzerland (n = 14).

The total number of isolates per country and the number of plasmids in the reference panel from countries in our study, as well as Italy and Switzerland, are indicated. The number of isolates harbouring an IncI1 replicon identified with PlasmidFinder are given, with the percentage with respect to the total number of isolates given in parenthesis. The distribution of IncI1 replicons by country for the 1,194 IncI1 replicon-positive isolates, are also shown. The isolates harbouring an IncI1 plasmid with > 98% match to the reference panel plasmids is given for each country.

**Figure 4:**
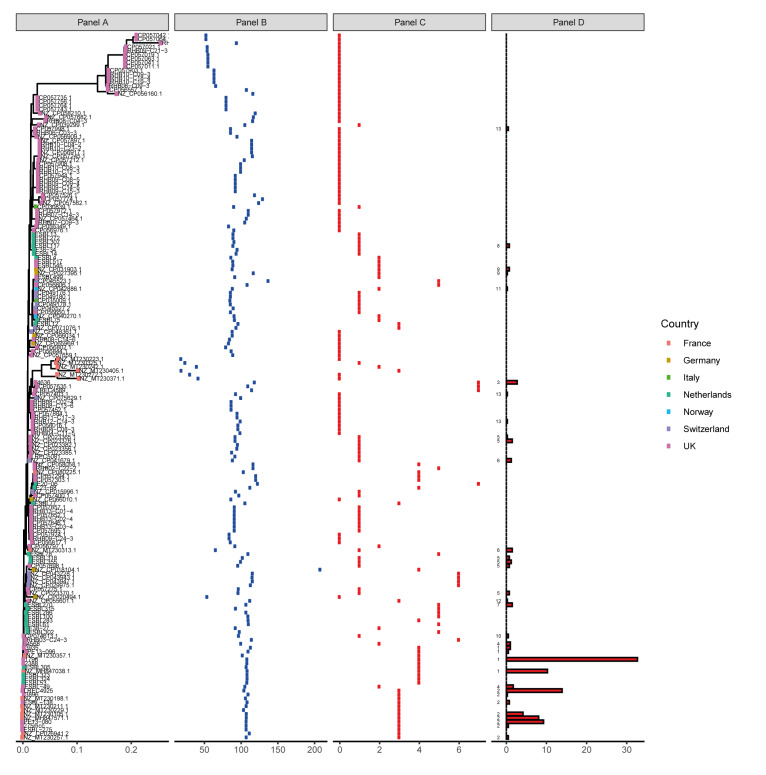
Phylogeny of the 181 IncI1-reference plasmids harboured by *Escherichia coli* isolates from France, Germany, the Netherlands, Norway, Spain, the United Kingdom, 2013–2020 (n =410)

Using a reference panel of 181 IncI1 plasmids and a BLASTn based method, the IncI1 plasmids for 410 isolates were identified (> 98% coverage). The remaining isolates (n = 784) with an IncI1 replicon did not show 98% identity, indicating the reference panel was not representative of the total diversity of IncI1 plasmid types circulating in these One Health compartments. The dendrogram produced from genome similarity analysis of the reference plasmids showed a clear difference in their genetic relatedness, with a number being more conserved and representing plasmids harboured by isolates in our dataset (Figure 4).

UK: United Kingdom.

Panel A. Phylogeny (Mash distance) of the reference plasmid set is presented.

Panel B. The length in kilobases of IncI1 reference plasmids is presented.

Panel C. Number of antimicrobial genes present in each reference plasmid.

Panel D. Distribution of *Escherichia coli* isolates with > 98% coverage and > 99% identity to the reference panel. The numbers on the left of the panel indicate the antimicrobial resistance profile index as per Supplementary Table S7.

The distribution of all isolates containing an IncI1 reference-like plasmid across the countries included is shown in Table 3. The occurrence of conserved IncI1 plasmids was lower in Spain, Norway and France (0.8%, 0.0% and 0.8%) compared with Germany and the Netherlands (3.4% and 6.2%) and the UK (17%). The authors have additionally shown in Supplementary Figure S4 these 410 isolates were distributed across the main isolate phylogeny, suggesting conserved plasmids had been transmitted horizontally in *E. coli* across several compartments and countries (Figure 3), although very few matched any IncI1 plasmid present in the human UTI isolates (phylogroup B2).

As shown in Supplementary Table S7, AMR profiles for the corresponding IncI1 plasmids in the 410 isolates indicated all profiles except three (profiles 10, 12 and 13) harboured AMR genes to ESCs, including ESBLs. Profile 12 harboured AMR genes to ampicillins and 13 harboured no AMR genes. The most common profile, profile 1, harboured several AMR genes, including ESBLs, and was present in 182 (44.4%) isolates from three countries (Germany, the Netherlands, and the UK) which is indicated in Supplementary Table S8. Supplementary Table S9 demonstrates that 83 were from pigs, 63 from poultry meat, 32 from poultry, three from cattle and one from pork. The second most common AMR profile, profile 2, also with multiple AMR genes, including ESBLs, was present in 153 (37.3%) isolates from two countries (UK and the Netherlands), with 79 from poultry meat, 59 from poultry and 15 from pigs. The most common reference-like plasmid present was 1796 which was isolated in 2015 from pork in the UK, with AMR profile 1. It was identified in 134 isolates from three countries: the UK, the Netherlands and Germany as shown in Supplementary Table S10; with Supplementary Table S11 showing the isolates were from poultry meat (n = 60), pigs (n = 43), poultry (n = 28), cattle (n = 2) and pork (n = 1). The second most common plasmid was LREC4925, also from the UK and isolated from poultry in 2020, with AMR profile 2. It was present in 57 isolates from the Netherlands (n = 2) and the UK (n = 55), mainly from poultry sources (poultry: n = 21; poultry meat: n = 35; pigs: n = 1).

## Discussion

Antimicrobial resistance is a global issue widely detected in bacteria from humans, animals, food and the environment. The ARDIG project aimed to detect AMR genes in *E. coli* isolated from different compartments and geographic regions, assessing their phylodynamic distribution and AMR plasmid transmission. Our study included more than double the number of isolates compared with the combined European–North American study on ESC *E. coli* (n = 1,818) [16].

The annual EFSA and ECDC reporting [10,25] has demonstrated differences in AMR distribution between European countries and different animal hosts, mainly attributed to antimicrobial usage [9]. Results from this extensive European WGS study, conferred with those findings. Further, by using WGS rather than minimum inhibitory concentration (MIC) data, we were able to detail several differences in AMR mechanisms. For example, we noted that only human isolates harboured AmpC promoter mutations at -32 (T > A) and carbapenem resistance genes, although in low numbers, were not detected in other hosts in this study, but have occasionally been reported in *E. coli* from European livestock [35]. As the use of carbapenem is not permitted in livestock in Europe, the latter was not surprising, but the significance of the AmpC promoter mutations specific to human isolates is not yet known. Also, presence of *tet*(X6), which confers resistance to tigecycline, was unexpectedly found in three cattle isolates from the same farm in the Netherlands adding to the growing list of livestock isolates detected with tigecycline resistance in Europe [11], suggesting this resistance may be more widespread, and possibly affect therapeutic outcomes in future.

Phylogenetic analysis mostly clustered *E. coli* according to phylogroups, with three phylogroups, B2, D and F, harbouring most of the human isolates. Phylogroup B2 has been associated with virulence [36,37], so their presence in clinical isolates was unsurprising. The phylogroup D of *E. coli* has previously been associated with virulence in animals, but to a lesser degree in humans [38], and we are not aware of phylogroup F being reported from humans before. Food animal isolates were distributed more widely, although less were present within human dominant phylogroups. The separation of most pathogenic and commensal *E. coli* suggests distinct evolution, although these may also be due to food animal isolates being from healthy animals. There is also suggestion of some co-evolution/transmission between compartments as several human *E. coli* were interspersed within livestock clades and vice versa.

Indeed, we inferred transmission between compartments from low SNP distances. Even though we could not infer directionality from the present data, except instances between poultry and poultry meat, it demonstrated vertical TEs between different One Health compartments and European countries occur. Although these vertical TEs were detected infrequently from our current dataset, the abundance that these occur in will require further in-depth sampling. We also noted that the SNP distances between linked isolates were generally > 20 SNPs, previously *E. coli* clones from a farm in a longitudinal study over 18 months has defined clones as ≤ 20 SNPs [22]. Therefore, this number needs revising when considering more distal (temporal and/or distant) events where evolutionary rates will be higher, as *E. coli* mutation rates are between 2.26 × 10^−7^ and 3.0 × 10^−6^ substitutions per base pair per year [39,40] so higher substitution rates are expected within *E. coli* genomes over the 7-year period of our study. It is also worth noting that this rate is expected to be higher than during outbreaks, which typically focus on tracing transmission of a single pathogenic clone/ST over weeks or months and are usually based on low SNP differences (e.g. < 10) [41]. Further, we hope studies like ours help provide evidence to build a consensus on what is the appropriate size of a cluster and the types of information it should contain for assessing a TE.

Detection of conserved IncI1 AMR plasmids, in *E. coli* from different hosts through vertical transmission, was limited. Only three different vertical transmission clusters with IncI1 AMR plasmids were detected where plasmids harbouring ESBL genes were present. It suggested plasmid transmission through bacterial replication was not widespread. However, a novel method using draft genomes, demonstrated there was widespread horizontal transmission of conserved AMR plasmids in isolates from different countries and livestock hosts. For example, several reference IncI1 AMR plasmids from *E. coli* isolated from livestock in Oxfordshire [33] were conserved and detected in isolates across the UK and Europe, although their origin remains unknown.

Surprisingly, very few of the 117 human *E. coli* with IncI1 showed matches to the IncI1 reference plasmids, suggesting very few human isolates carried these variants despite 25 IncI1 reference plasmids being of human origin. Also, very few isolates in our dataset matched genomes of the 25 IncI1 reference plasmids from human isolates. Therefore, there is possibly a faster evolutionary rate and/or a greater diversity in plasmids harboured by the human isolates included in this study, and/or limited transmission between humans and the other compartments we studied. This could be because all our human isolates were of clinical origin and mainly from urinary tract infections, so more likely to be exposed to antimicrobials and evolving more quickly than those from healthy animals or animal products, which possibly had lower antimicrobial exposure, so plasmids may remain more stable due to less selective pressure. Alternatively, it might reflect a bias in our plasmid database, which was of European plasmids, as our aim was to determine AMR plasmid mediated dissemination in Europe. However, these results suggest a much wider diversity in plasmids and/or faster evolution in human isolates as modern human lifestyles include frequent travel and intermixing beyond Europe. We also noted that isolates from the UK and the Netherlands shared the same IncI1 plasmids more often than the other countries. This might be because most IncI1 plasmids in the reference panel were from these two countries.

Although there were several differences in the isolate sampling framework between countries and compartments, which was a limitation of this study, we nevertheless believe the results are representative of the *E. coli* populations from these compartments. This is because we noted that the human clinical isolates, which despite representing > 1,000 unique individuals from four countries sampled over 1 year, were phylogenetically more closely related than animal isolates; this could reflect human hospital-acquired infections. Similar numbers of animal isolates were included from national monitoring and longitudinal studies to each other, and to human isolates. We agree inclusion of multiple isolates from longitudinal sampling on the same farm could result in clonal populations and less diversity [42] than those collected for national monitoring from separate index farms. However, the poultry and pig samplings were performed over considerably long periods, representing multiple flocks/batches with thorough cleaning and disinfection in between livestock re-population. Similarly, most cattle isolates were collected from a large cross-sectional study [21], so there was considerable mixing of populations.

Also, we believe the difference in culture media used between humans and livestock samples did not notably affect our ability to compare AMR. Human isolates were from clinical samples and harboured a large diversity of predicted resistances including to MIAs, although isolated from non-selective plates. In contrast, commensal *E. coli* from healthy animals harboured less resistance genes to MIAs so a combination of *E. coli* from both selective and non-selective plates were able to detect AMR, including to MIAs which may be present at low level. Importantly, despite differences in sampling framework (diseased humans vs healthy livestock), and isolation media (non-selective vs selective) a plethora of AMR genes were detected in isolates from all compartments. However, comparison between countries and sources was made for AMR gene occurrence rather than prevalence due to isolates being samples of convenience.

## Conclusion

In conclusion, this study, based on a large number of samples, advances our knowledge on AMR genes present in different One Health compartments in six European countries, and shows that they can be transferred both vertically and horizontally. Crucially, our study suggests that the stability, evolution and/or diversity of AMR plasmids harbouring resistance to MIAs may differ between humans and other compartments, which likely resulted from differing environments. These could include differences in authorised drug classes for human and veterinary usage, varying lifestyles or environments and contact with chemical substances. As plasmids are important drivers for AMR, affecting AMR risk and mitigation policies in Europe, and worldwide, factors influencing their stability and dissemination warrants further study. Future surveillance and monitoring of AMR could be improved through a more comprehensive sampling framework considering differences in the population (clinical human vs healthy animal samples) and sampling strategies (passive monitoring vs randomised sampling) which are planned over a longer period to help determine AMR directionality. Also, assessing travel history, chemical and environmental exposures of individuals/livestock will lead to a better understanding of AMR evolution and transmission pathways, and hence its control.

## Supplementary Data

1233000 Supplementary Material 1

## Supplementary Data

294000 Supplementary Material 2

## Supplementary Data

79000 Supplementary Material 3

## Supplementary Data

34000 Supplementary Material 4

## Supplementary Data

31000 Supplementary Material 5
