# Supplementary material for: Escherichia coli from six European countries reveals differences in profile and distribution of critical antimicrobial resistance determinants within One Health compartments, 2013 to 2020
Source: Euro Surveill. 2024 Nov 21;29(47):2400295. doi: 10.2807/1560-7917.ES.2024.29.47.2400295 (PMC11583308; doi:10.2807/1560-7917.ES.2024.29.47.2400295)
Supplement: Supplementary Material 1 [file 24-00295_ANJUM_Supplementary_Figures_Tables_S2-S3_S7-S11.pdf]

# A study of *Escherichia coli* collected between 2013-2020 from six European countries reveals differences in profile and distribution of critical antimicrobial resistance determinants within One-Health compartments.

# Supplementary Methods, Figures and Tables

**Disclaimer:** This supplementary material is hosted by Eurosurveillance as supporting information alongside this article, on behalf of the authors, who remain responsible for the accuracy and appropriateness of the content. The same standards for ethics, copyright, attributions and permissions as for the article apply. Supplements are not edited by Eurosurveillance and the journal is not responsible for the maintenance of any links or email addresses provided therein

## IncI1 plasmid method evaluation

The IncI1 plasmid identification method was validated using the assemblies generated from short-read WGS for 46 isolates that were already known to contain circularised IncI1 plasmids from hybrid sequencing [32], which have also been included in the reference set of plasmids. The assemblies used the same 3 quality conditions as the 3475 isolates used in this study: a) removal of contigs shorter than 300bp, b) the total number of contigs left is smaller or equal to 280 and c) average length of the contigs left is greater than 18000 bp. The method assigned the correct IncI1 plasmid in the reference panel for 40 out of the 46 assemblies used (80%), with a percentage identity greater than 98%. For the 6 assemblies that were not assigned to their corresponding plasmid as the best match, one isolate had a 99% identity to its corresponding IncI1 plasmid but with best match to a different plasmid (99.95% identity); 2 isolates had 95% identity to the IncI1 plasmid with best match to another plasmid (99.5% identity); a further two isolates had 85% identity to the IncI1 plasmid with 99.6% identity to another plasmid; and one isolate had 39% identity to the IncI1 plasmid, with 45% identity to another. Further investigation using the available long read assemblies, showed artifacts on the assemblies for these six isolates as the cause of failure, with the assembler tool joining part of the plasmid and chromosomal DNA into the same contig. Since such contigs did not match the reference plasmid DNA with >99% identity, the method discarded the contig lowering the highest percentage match with the plasmid.

# Supplementary Figures

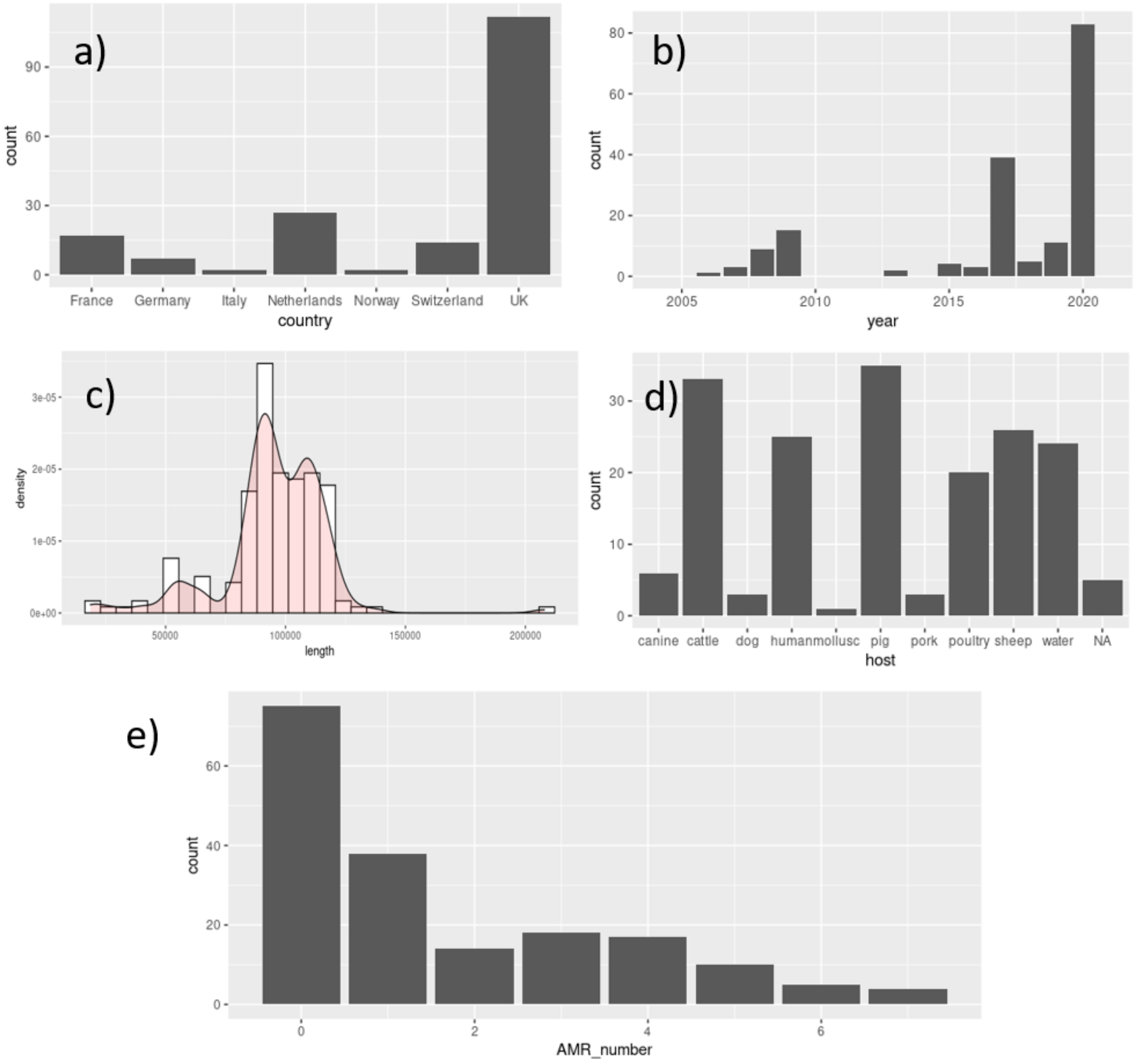


**Figure S1:** Background of the IncI1 plasmid genomes included in the reference panel by: a) country of isolation; b) year of isolation; c) plasmid length; d) animal host/source; d) number of AMR genes.

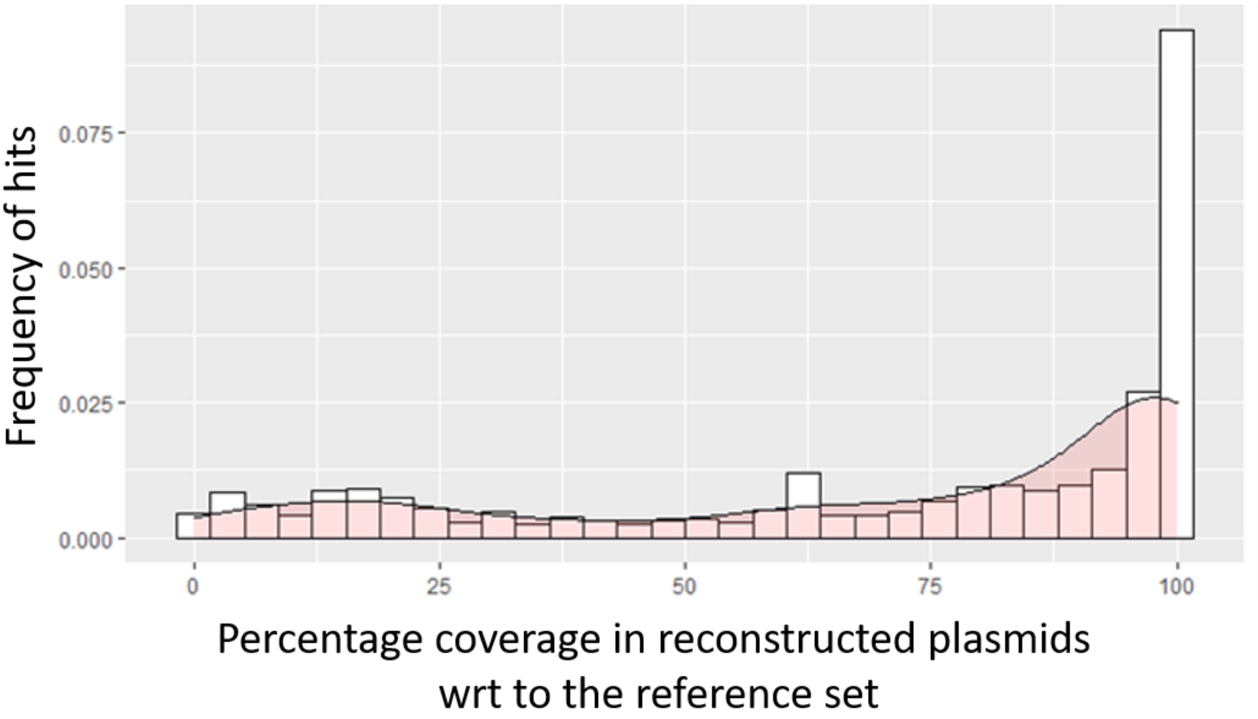


**Figure S2:** Distribution of the highest hit to the reference panel for the 1194 isolates with an IncI1 replicon. The highest hit for an isolate is defined by the BLASTn score when sequences in the assembled contigs of isolates are compared with sequences in the IncI1 plasmid reference panel. The highest percentage coverage for assemblies present in each isolate showing >99% identity to the reference plasmids was plotted. Only isolates with contigs showing >98% coverage to any reference plasmid was included in this study.

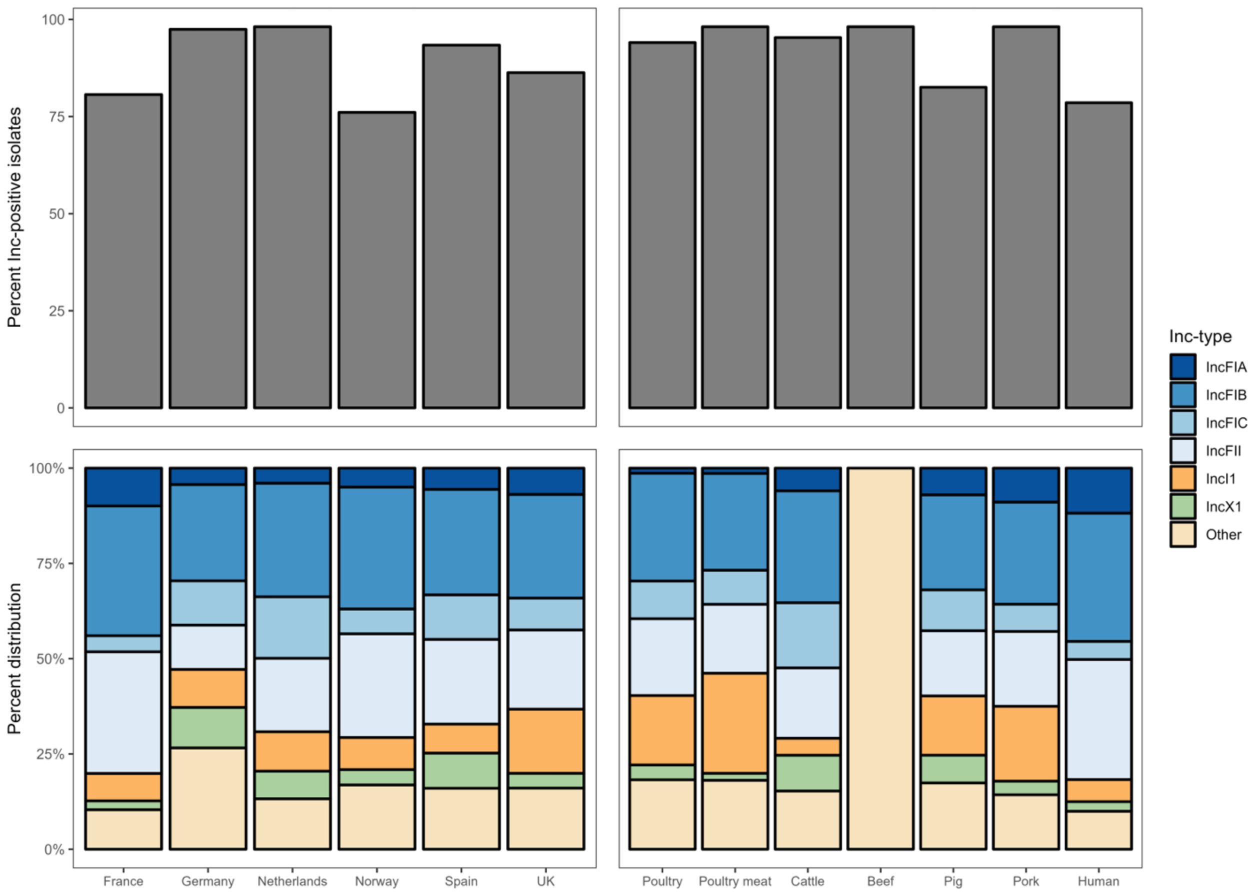


**Figure S3:** Distribution of different plasmid Inc-types identified with PlasmidFinder present in 3745 *E. coli*, stratified on country and host/sample type.

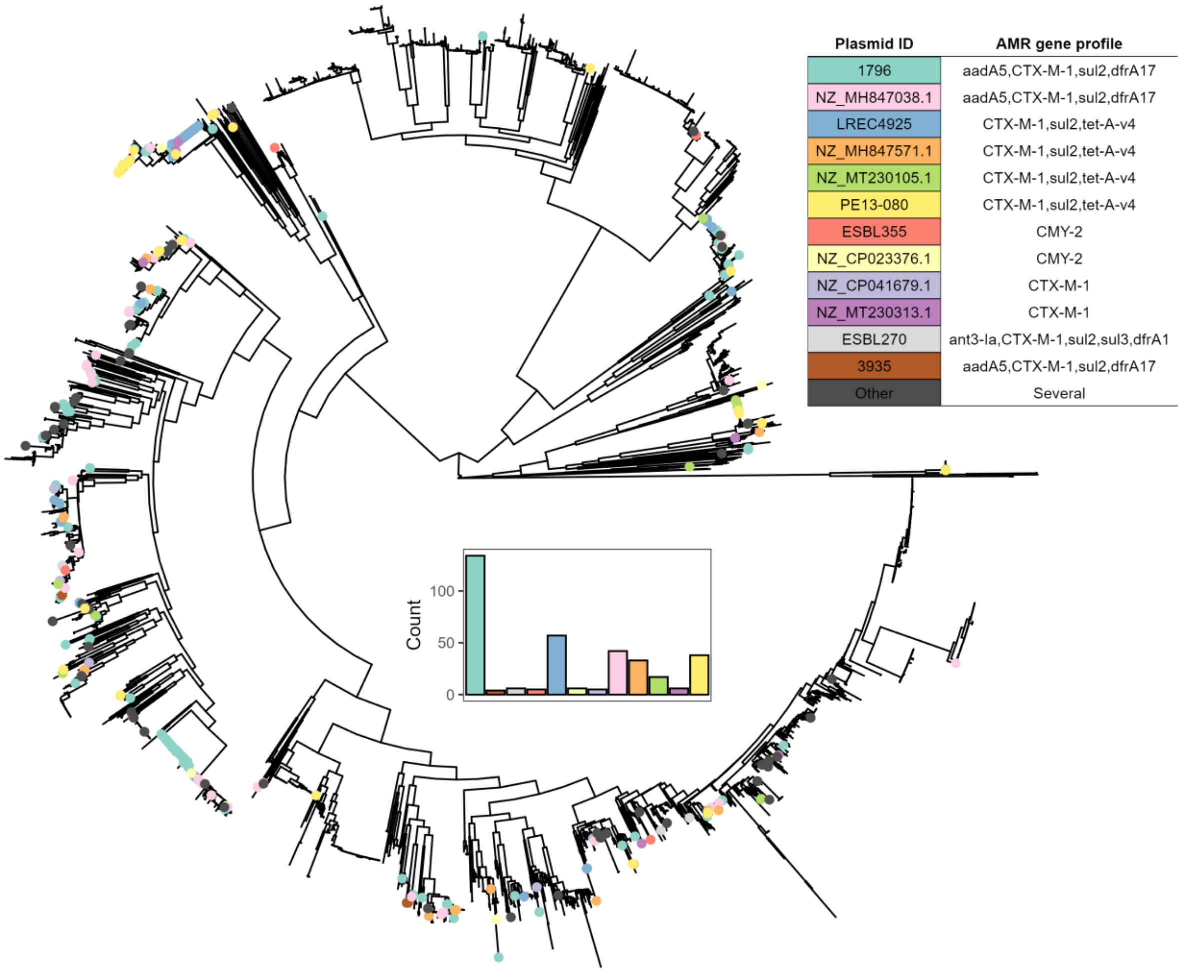


**Figure S4:** Maximum likelihood phylogeny based on an alignment of 3103 core genes from all included isolates in this study, with the 410 identified IncI1-positive isolates highlighted. Plasmids presented with a colour represent the major groups of reference plasmids identified in the 410 IncI1-positive isolates. These plasmids were identified in at least one isolate from a different country than the reference plasmid origin. The bar plot presents the number of isolates with each respective IncI1 plasmid.

## Supplementary Tables

**Table S2:** DNA extraction and sequencing methods.

| Country | DNA isolation | Library method | Sequenced |
|---|---|---|---|
| The Netherlands | Qiagen DNeasy Blood & Tissue kit | Nextera Flex kit | Illumina Miseq<br>Illumina Hiseq<br>Illumina Nextseq |
| Norway | QIAamp DNA mini kit | Nextera DNA Flex | Illumina MiSeq<br>Illumina HiSeq |
| UK | KingFisher | Nextera DNA Flex | Illumina Nextseq |
| France | QIAamp DNA mini kit | Nextera DNA kit or NEBNext Ultra II FS DNA Library prep | Illumina Nextseq |
| Germany | Invitrogen Purelink DNA mini kit | Nextera DNA Flex | Illumina Nextseq |
| Spain | QIAamp DNA mini kit | Nextera DNA XT | Illumina MiSeq |

**Table S3:** Characteristics and origin of the IncI1 reference plasmids conserved among isolates of *Escherichia coli* from France, Germany, the Netherlands, Norway, Switzerland, and the United Kingdom. Distribution among the 410 IncI1-positive isolates in our study is presented in the last two columns.

| Plasmid ID | Circular | pMLST | Size (bp) | Source | Year | Origin | Country | AMR profile | Distribution | |
|---|---|---|---|---|---|---|---|---|---|---|
| | | | | | | | | | Number of isolates | % |
| 1796 | Yes | 3 | 108,489 | Pork | 2015 | APHA | UK | 1 | 134 | 32.68 |
| LREC4925 | Yes | 3 | 104,515 | Poultry | 2020 | APHA | UK | 2 | 57 | 13.9 |
| NZ_MH847038.1 | Yes | 220[a] | 108,813 | Pig | 2020 | NCBI | FR | 1 | 42 | 10.24 |
| PE13–080 | Yes | 3 | 107,495 | Pig | 2013 | APHA | UK | 2 | 38 | 9.27 |
| NZ_MH847571.1 | Yes | 220[a] | 107,715 | Pig | 2020 | NCBI | FR | 2 | 33 | 8.05 |
| NZ_MT230105.1 | Yes | 3 | 107,737 | Water | 2020 | NCBI | FR | 2 | 17 | 4.15 |
| 4636 | Yes | 80 | 118,642 | Pig | 2019 | APHA | UK | 3 | 11 | 2.68 |
| ESBL-49 | Yes | 3 | 105,788 | Pig | 2017 | APHA | UK | 4 | 7 | 1.71 |
| ESBL270 | No | 7 | 107,175 | Poultry | 2007 | Smith | NL | 7 | 6 | 1.46 |
| NZ_CP023376.1 | Yes | 2 | 92,420 | Canine | 2017 | NCBI | UK | 5 | 6 | 1.46 |
| NZ_MT230313.1 | Yes | Unknown | 65,694 | Water | 2020 | NCBI | FR | 6 | 6 | 1.46 |
| ESBL355 | No | 12 | 99,885 | Poultry | 2007 | Smith | NL | 5 | 5 | 1.22 |
| NZ_CP041679.1 | Yes | 58 | 88,891 | Human | 2019 | NCBI | CH | 6 | 5 | 1.22 |
| 3935 | Yes | 3 | 113,383 | Poultry | 2018 | APHA | UK | 1 | 4 | 0.98 |
| 4568 | Yes | 108 | 100,114 | Pork | 2019 | APHA | UK | 4 | 4 | 0.98 |
| CP057698.1 | Yes | 12 | 96,066 | Pig | 2020 | NCBI | UK | 5 | 3 | 0.73 |
| ESBL117 | No | 36 | 89,503 | Human | 2009 | Smith | NL | 8 | 3 | 0.73 |
| ESBL-138 | Yes | 3 | 108,782 | Pig | 2015 | APHA | UK | 2 | 3 | 0.73 |
| ESBL318 | No | 12 | 102,363 | Poultry | 2008 | Smith | NL | 5 | 3 | 0.73 |
| NZ_CP023370.1 | Yes | 43 | 96,402 | Canine | 2017 | NCBI | UK | 5 | 3 | 0.73 |
| NZ_CP031903.1 | Yes | 31 | 88,545 | Human | 2019 | NCBI | DE | 9 | 3 | 0.73 |
| 275mcc | Yes | 3 | 107,495 | Pig | 2015 | APHA | UK | 2 | 2 | 0.49 |
| CP057998.1 | Yes | 134 | 86,278 | Sheep | 2020 | NCBI | UK | 13 | 2 | 0.49 |

| | | | | | | | | | | |
|---|---|---|---|---|---|---|---|---|---|---|
| CP074613.1 | Yes | 3 | 97,119 | Unknown | 2021 | NCBI | UK | 10 | 2 | 0.49 |
| NZ_MT230257.1 | Yes | 3 | 107,610 | Water | 2020 | NCBI | FR | 2 | 2 | 0.49 |
| PE13–096 | Yes | 3[a] | 110,096 | Pig | 2013 | APHA | UK | 1 | 2 | 0.49 |
| 3896 | Yes | 3[a] | 110,139 | Poultry | 2018 | APHA | UK | 2 | 1 | 0.24 |
| CP057403.1 | Yes | Unknown | 93,112 | Pig | 2020 | NCBI | UK | 14 | 1 | 0.24 |
| NZ_CP023365.1 | Yes | 2 | 92,421 | Canine | 2017 | NCBI | UK | 5 | 1 | 0.24 |
| NZ_CP027395.1 | No | Unknown | 117,229 | Human | 2018 | NCBI | DE | 9 | 1 | 0.24 |
| NZ_CP042886.1 | Yes | 31 | 88,772 | Water | 2020 | NCBI | NO | 11 | 1 | 0.24 |
| NZ_CP055601.1 | Yes | Unknown | 112,380 | Pig | 2020 | NCBI | UK | 12 | 1 | 0.24 |
| RHB12-C14–3 | Yes | Unknown | 99,450 | Cattle | 2017 | APHA | UK | 13 | 1 | 0.24 |

[a]: The closest pMLST-profile but not an exact match

**Table S7:** AMR profiles for the 410 IncI1-plasmids identified in isolates through PlasmidFinder and with >98% match to the reference panel.

| Index | AMR profile |
|---|---|
| **1** | *aadA5*, $bla_{CTX-M-1}$, *sul2, dfrA17* |
| **2** | $bla_{CTX-M-1}$, *sul2, tet-A-v4* |
| **3** | *strA, strB,* $bla_{CTX-M-14}$, $bla_{LAP-2}$, *qnrS1*, *sul2*, *dfrA14* |
| **4** | $bla_{CTX-M-1}$, *sul2* |
| **5** | $bla_{CMY-2}$ |
| **6** | $bla_{CTX-M-1}$ |
| **7** | *ant(3'')-Ia,* $bla_{CTX-M-1}$, *sul2*, *sul3*, *dfrA1* |
| **8** | $bla_{TEM-52c}$ |
| **9** | $bla_{CTX-M-15}$, $bla_{TEM-1b}$ |
| **10** | *sul2* |
| **11** | $bla_{CTX-M-15}$, *qnrS1* |
| **12** | $bla_{TEM-1b}$, *sul2, dfrA1* |
| **13** | No AMR genes |

**Table S8:** Number of samples associated with each of the 13 AMR profiles found for the 410 positive samples containing IncI1 plasmids and their distribution across countries. Number of isolates per country are indicated by #; Norway is excluded as there were no matches at >98% coverage and >99% identity. For every country, column *%_c* indicates as percentage the numbers of positive isolates w.r.t. the total positives for that AMR profile in column two and column *%_p* indicates as percentage the number of positive isolates w.r.t. the number of IncI1 positive isolates (plasmid finder + 98% coverage) from each country as described in Table 3.

| | | | **Netherlands** | | | **UK** | | | **France** | | | **Spain** | | | **Germany** | | |
|---|---|---|---|---|---|---|---|---|---|---|---|---|---|---|---|---|---|
| **AMR profile index** | **counts** | **% (of 410)** | **#** | **%_c** | **%_p** | **#** | **%_c** | **%_p** | **#** | **%_c** | **%_p** | **#** | **%_c** | **%_p** | **#** | **%_c** | **%_p** |
| **1** | 182 | 44.39 | 7 | 3.85 | 19.44 | 173 | 95.05 | 47.4 | 0 | 0 | 0 | 0 | 0 | 0 | 2 | 1.1 | 50 |
| **2** | 153 | 37.32 | 12 | 7.84 | 33.33 | 141 | 92.16 | 38.63 | 0 | 0 | 0 | 0 | 0 | 0 | 0 | 0 | 0 |
| **3** | 11 | 2.68 | 0 | 0 | 0 | 11 | 100 | 3.01 | 0 | 0 | 0 | 0 | 0 | 0 | 0 | 0 | 0 |
| **4** | 11 | 2.68 | 0 | 0 | 0 | 11 | 100 | 3.01 | 0 | 0 | 0 | 0 | 0 | 0 | 0 | 0 | 0 |
| **5** | 21 | 5.12 | 7 | 33.33 | 19.44 | 13 | 61.9 | 3.56 | 0 | 0 | 0 | 1 | 4.76 | 33.33 | 0 | 0 | 0 |
| **6** | 11 | 2.68 | 4 | 36.36 | 11.11 | 5 | 45.45 | 1.37 | 1 | 9.09 | 50 | 0 | 0 | 0 | 1 | 9.09 | 25 |
| **7** | 6 | 1.46 | 2 | 33.33 | 5.56 | 4 | 66.67 | 1.1 | 0 | 0 | 0 | 0 | 0 | 0 | 0 | 0 | 0 |
| **8** | 3 | 0.73 | 3 | 100 | 8.33 | 0 | 0 | 0 | 0 | 0 | 0 | 0 | 0 | 0 | 0 | 0 | 0 |
| **9** | 4 | 0.98 | 0 | 0 | 0 | 4 | 100 | 1.1 | 0 | 0 | 0 | 0 | 0 | 0 | 0 | 0 | 0 |
| **10** | 2 | 0.49 | 0 | 0 | 0 | 2 | 100 | 0.55 | 0 | 0 | 0 | 0 | 0 | 0 | 0 | 0 | 0 |
| **11** | 1 | 0.24 | 1 | 100 | 2.78 | 0 | 0 | 0 | 0 | 0 | 0 | 0 | 0 | 0 | 0 | 0 | 0 |
| **12** | 1 | 0.24 | 0 | 0 | 0 | 0 | 0 | 0 | 0 | 0 | 0 | 1 | 100 | 33.33 | 0 | 0 | 0 |
| **13** | 4 | 0.98 | 0 | 0 | 0 | 1 | 25 | 0.27 | 1 | 25 | 50 | 1 | 25 | 33.33 | 1 | 25 | 25 |
| **Total** | 410 | | 36 | | | 365 | | | 2 | | | 3 | | | 4 | | |

**Table S9:** Number of samples associated with each of the 13 AMR profiles found for the 410 positive samples for IncI1 plasmid and the distribution across the host species. The number of isolates per host-group are indicated by #.

| | | | Poultry | | Poultry meat | | Pork | | Human | | Cattle | | Pig | |
|---|---|---|---|---|---|---|---|---|---|---|---|---|---|---|
| **AMR profile index** | **counts** | **% (of 410)** | **#** | **%** | **#** | **%** | **#** | **%** | **#** | **%** | **#** | **%** | **#** | **%** |
| **1** | 182 | 44.39 | 32 | 17.6 | 63 | 34.6 | 1 | 0.6 | 0 | 0 | 3 | 1.65 | 83 | 46 |
| **2** | 153 | 37.32 | 59 | 38.6 | 79 | 51.6 | 0 | 0 | 0 | 0 | 0 | 0 | 15 | 9.8 |
| 3 | 11 | 2.68 | 0 | 0 | 0 | 0 | 0 | 0 | 0 | 0 | 0 | 0 | 11 | 100 |
| 4 | 11 | 2.68 | 1 | 9.1 | 0 | 0 | 2 | 18 | 0 | 0 | 0 | 0 | 8 | 73 |
| 5 | 21 | 5.12 | 7 | 33.3 | 3 | 14.3 | 1 | 4.8 | 0 | 0 | 2 | 9.52 | 8 | 38 |
| 6 | 11 | 2.68 | 2 | 18.2 | 1 | 9.09 | 0 | 0 | 1 | 9.1 | 3 | 27.29 | 4 | 36 |
| 7 | 6 | 1.46 | 0 | 0 | 0 | 0 | 0 | 0 | 0 | 0 | 0 | 0 | 6 | 100 |
| 8 | 3 | 0.73 | 2 | 66.7 | 0 | 0 | 0 | 0 | 0 | 0 | 1 | 33.3 | 0 | 0 |
| 9 | 4 | 0.98 | 0 | 0 | 0 | 0 | 0 | 0 | 0 | 0 | 0 | 0 | 4 | 100 |
| 10 | 2 | 0.49 | 0 | 0 | 0 | 0 | 0 | 0 | 0 | 0 | 0 | 0 | 2 | 100 |
| 11 | 1 | 0.24 | 0 | 0 | 0 | 0 | 0 | 0 | 0 | 0 | 1 | 100 | 0 | 0 |
| 12 | 1 | 0.24 | 0 | 0 | 0 | 0 | 0 | 0 | 1 | 100 | 0 | 0 | 0 | 0 |
| 13 | 4 | 0.98 | 1 | 25 | 0 | 0 | 0 | 0 | 1 | 25 | 0 | 0 | 0 | 0 |

**Table S10**: Number of samples associated with each of the plasmids in the reference set. The sum of the counts is 410. For every country, column *%_c* indicates as percentage the number of isolates w.r.t. the total positive isolates for a particular plasmid type (Table 1). For every country, column *%_p* indicates as percentage the number of isolates w.r.t. the number of IncI1 positive isolates from each country as described in Table 3 (column IncI1 plasmid with >98% match with ref panel).

| | | *Germany* | | | *Netherlands* | | | *Spain* | | | *UK* | | | *France* | | | |
|---|---|---|---|---|---|---|---|---|---|---|---|---|---|---|---|---|---|
| ***plasmid ID*** | ***country*** | ***#*** | ***%_p*** | ***%_c*** | ***#*** | ***%_p*** | ***%_c*** | ***#*** | ***%_p*** | ***%_c*** | ***#*** | ***%_p*** | ***%_c*** | ***#*** | ***%_p*** | ***%_c*** | **AMR profile** |
| ***1796*** | ***UK*** | *1* | *0.7* | *25* | *3* | *2.2* | *8.3* | *0* | *0* | *0* | *130* | *97* | *35.6* | *0* | *0* | *0* | **1** |
| ***LREC4925*** | ***UK*** | *0* | *0* | *0* | *2* | *3.5* | *5.6* | *0* | *0* | *0* | *55* | *96.5* | *15.1* | *0* | *0* | *0* | **2** |
| ***NZ_MH847038.1*** | ***France*** | *0* | *0* | *0* | *4* | *9.5* | *11.1* | *0* | *0* | *0* | *38* | *90.5* | *10.4* | *0* | *0* | *0* | **1** |
| ***PE13-080*** | ***UK*** | *0* | *0* | *0* | *2* | *5.3* | *5.6* | *0* | *0* | *0* | *36* | *94.7* | *9.9* | *0* | *0* | *0* | **2** |
| ***NZ_MH847571.1*** | ***France*** | *0* | *0* | *0* | *6* | *18.2* | *16.7* | *0* | *0* | *0* | *27* | *81.8* | *7.4* | *0* | *0* | *0* | **2** |
| ***NZ_MT230105.1*** | ***France*** | *0* | *0* | *0* | *0* | *0* | *0* | *0* | *0* | *0* | *17* | *100* | *4.7* | *0* | *0* | *0* | **2** |
| ***4636*** | ***UK*** | *0* | *0* | *0* | *0* | *0* | *0* | *0* | *0* | *0* | *11* | *100* | *3* | *0* | *0* | *0* | **3** |
| ***ESBL-49*** | ***UK*** | *0* | *0* | *0* | *0* | *0* | *0* | *0* | *0* | *0* | *7* | *100* | *1.9* | *0* | *0* | *0* | **4** |
| ***ESBL270*** | ***Nether-lands*** | *0* | *0* | *0* | *2* | *33.3* | *5.6* | *0* | *0* | *0* | *4* | *66.7* | *1.1* | *0* | *0* | *0* | **7** |
| ***NZ_CP023376.1*** | ***UK*** | *0* | *0* | *0* | *3* | *50* | *8.3* | *0* | *0* | *0* | *3* | *50* | *0.8* | *0* | *0* | *0* | **5** |
| ***NZ_MT230313.1*** | ***France*** | *0* | *0* | *0* | *2* | *33.3* | *5.6* | *0* | *0* | *0* | *4* | *66.7* | *1.1* | *0* | *0* | *0* | **6** |
| ***ESBL355*** | ***Nether-lands*** | *0* | *0* | *0* | *1* | *20* | *2.8* | *0* | *0* | *0* | *4* | *80* | *1.1* | *0* | *0* | *0* | **5** |
| ***NZ_CP041679.1*** | ***Switzer-land*** | *1* | *20* | *25* | *2* | *40* | *5.6* | *0* | *0* | *0* | *1* | *20* | *0.3* | *1* | *20* | *50* | **6** |
| ***3935*** | ***UK*** | *1* | *25* | *25* | *0* | *0* | *0* | *0* | *0* | *0* | *3* | *75* | *0.8* | *0* | *0* | *0* | **1** |
| ***4568*** | ***UK*** | *0* | *0* | *0* | *0* | *0* | *0* | *0* | *0* | *0* | *4* | *100* | *1.1* | *0* | *0* | *0* | **4** |
| ***CP057698.1*** | ***UK*** | *0* | *0* | *0* | *3* | *100* | *8.3* | *0* | *0* | *0* | *0* | *0* | *0* | *0* | *0* | *0* | **5** |
| ***ESBL117*** | ***Nether-lands*** | *0* | *0* | *0* | *3* | *100* | *8.3* | *0* | *0* | *0* | *0* | *0* | *0* | *0* | *0* | *0* | **8** |
| ***ESBL-138*** | ***UK*** | *0* | *0* | *0* | *0* | *0* | *0* | *0* | *0* | *0* | *3* | *100* | *0.8* | *0* | *0* | *0* | **2** |
| ***ESBL318*** | ***Nether-lands*** | *0* | *0* | *0* | *0* | *0* | *0* | *1* | *33.3* | *33.3* | *2* | *66.7* | *0.5* | *0* | *0* | *0* | **5** |
| ***NZ_CP023370.1*** | ***UK*** | *0* | *0* | *0* | *0* | *0* | *0* | *0* | *0* | *0* | *3* | *100* | *0.8* | *0* | *0* | *0* | **5** |
| ***NZ_CP031903.1*** | ***Germany*** | *0* | *0* | *0* | *0* | *0* | *0* | *0* | *0* | *0* | *3* | *100* | *0.8* | *0* | *0* | *0* | **9** |
| ***275mcc*** | ***UK*** | *0* | *0* | *0* | *0* | *0* | *0* | *0* | *0* | *0* | *2* | *100* | *0.5* | *0* | *0* | *0* | **2** |
| ***CP057998.1*** | ***UK*** | *0* | *0* | *0* | *0* | *0* | *0* | *1* | *50* | *33.3* | *0* | *0* | *0* | *1* | *50* | *50* | **13** |
| ***CP074613.1*** | ***UK*** | *0* | *0* | *0* | *0* | *0* | *0* | *0* | *0* | *0* | *2* | *100* | *0.5* | *0* | *0* | *0* | **10** |
| ***NZ_MT230257.1*** | ***France*** | *0* | *0* | *0* | *1* | *50* | *2.8* | *0* | *0* | *0* | *1* | *50* | *0.3* | *0* | *0* | *0* | **2** |
| ***PE13-096*** | ***UK*** | *0* | *0* | *0* | *0* | *0* | *0* | *0* | *0* | *0* | *2* | *100* | *0.5* | *0* | *0* | *0* | **1** |
| ***3896*** | ***UK*** | *0* | *0* | *0* | *1* | *100* | *2.8* | *0* | *0* | *0* | *0* | *0* | *0* | *0* | *0* | *0* | **2** |
| ***CP057403.1*** | ***UK*** | *0* | *0* | *0* | *0* | *0* | *0* | *0* | *0* | *0* | *1* | *100* | *0.3* | *0* | *0* | *0* | **14** |
| ***NZ_CP023365.1*** | ***UK*** | *0* | *0* | *0* | *0* | *0* | *0* | *0* | *0* | *0* | *1* | *100* | *0.3* | *0* | *0* | *0* | **5** |
| ***NZ_CP027395.1*** | ***Germany*** | *0* | *0* | *0* | *0* | *0* | *0* | *0* | *0* | *0* | *1* | *100* | *0.3* | *0* | *0* | *0* | **9** |
| ***NZ_CP042886.1*** | ***Norway*** | *0* | *0* | *0* | *1* | *100* | *2.8* | *0* | *0* | *0* | *0* | *0* | *0* | *0* | *0* | *0* | **11** |
| ***NZ_CP055601.1*** | ***UK*** | *0* | *0* | *0* | *0* | *0* | *0* | *1* | *100* | *33.3* | *0* | *0* | *0* | *0* | *0* | *0* | **12** |
| ***RHB12-C14-3*** | ***UK*** | *1* | *100* | *25* | *0* | *0* | *0* | *0* | *0* | *0* | *0* | *0* | *0* | *0* | *0* | *0* | **13** |

**Table S11.** Percent distribution of the reference IncI1 plasmids among the 410 samples positive for IncI1-plasmids using the BLAST method. The data is stratified on host species, presented as number of isolates (#) and percent occurrence (%) with respect to the total number of isolates harbouring the respective plasmid.

| | | Poultry | | Poultry Meat | | Pork | | Human | | Cattle | | Pig | | |
|---|---|---|---|---|---|---|---|---|---|---|---|---|---|---|
| **Assigned plasmid ID** | **Plasmid host** | **#** | **%** | **#** | **%** | **#** | **%** | **#** | **%** | **#** | **%** | **#** | **%** | **AMR profile** |
| **1796** | **pork** | 28 | 20.89 | 60 | 44.78 | 1 | 0.75 | 0 | 0 | 2 | 1.5 | 43 | 32.09 | **1** |
| **LREC4925** | **poultry** | 21 | 36.84 | 35 | 61.4 | 0 | 0 | 0 | 0 | 0 | 0 | 1 | 1.75 | **2** |
| **NZ_MH847038.1** | **pig** | 3 | 7.14 | 1 | 2.38 | 0 | 0 | 0 | 0 | 1 | 2.38 | 37 | 88.1 | **1** |
| **PE13-080** | **pig** | 8 | 21.05 | 20 | 52.63 | 0 | 0 | 0 | 0 | 0 | 0 | 10 | 26.32 | **2** |
| **NZ_MH847571.1** | **pig** | 16 | 48.48 | 15 | 45.45 | 0 | 0 | 0 | 0 | 0 | 0 | 2 | 6.06 | **2** |
| **NZ_MT230105.1** | **water** | 10 | 58.82 | 7 | 41.18 | 0 | 0 | 0 | 0 | 0 | 0 | 0 | 0 | **2** |
| **4636** | **pig** | 0 | 0 | 0 | 0 | 0 | 0 | 0 | 0 | 0 | 0 | 11 | 100 | **3** |
| **ESBL-49** | **pig** | 1 | 14.29 | 0 | 0 | 0 | 0 | 0 | 0 | 0 | 0 | 6 | 85.71 | **4** |
| **ESBL270** | **poultry** | 0 | 0 | 0 | 0 | 0 | 0 | 0 | 0 | 0 | 0 | 6 | 100 | **7** |
| **NZ_CP023376.1** | **canine** | 3 | 50 | 0 | 0 | 1 | 16.7 | 0 | 0 | 0 | 0 | 2 | 33.33 | **5** |
| **NZ_MT230313.1** | **water** | 2 | 33.33 | 1 | 16.67 | 0 | 0 | 0 | 0 | 0 | 0 | 3 | 50 | **6** |
| **ESBL355** | **poultry** | 3 | 60 | 1 | 20 | 0 | 0 | 0 | 0 | 0 | 0 | 1 | 20 | **5** |
| **NZ_CP041679.1** | **human** | 0 | 0 | 0 | 0 | 0 | 0 | 1 | 20 | 3 | 60 | 1 | 20 | **6** |
| **3935** | **poultry** | 1 | 25 | 2 | 50 | 0 | 0 | 0 | 0 | 0 | 0 | 1 | 25 | **1** |
| **4568** | **pork** | 0 | 0 | 0 | 0 | 2 | 50 | 0 | 0 | 0 | 0 | 2 | 50 | **4** |
| **CP057698.1** | **pig** | 1 | 33.3 | 0 | 0 | 0 | 0 | 0 | 0 | 2 | 66.7 | 0 | 0 | **5** |
| **ESBL117** | **human** | 2 | 66.67 | 0 | 0 | 0 | 0 | 0 | 0 | 1 | 33.3 | 0 | 0 | **8** |
| **ESBL-138** | **pig** | 0 | 0 | 2 | 66.67 | 0 | 0 | 0 | 0 | 0 | 0 | 1 | 33.33 | **2** |
| **ESBL318** | **poultry** | 0 | 0 | 2 | 66.67 | 0 | 0 | 0 | 0 | 0 | 0 | 1 | 33.33 | **5** |
| **NZ_CP023370.1** | **canine** | 0 | 0 | 0 | 0 | 0 | 0 | 0 | 0 | 0 | 0 | 3 | 100 | **5** |
| **NZ_CP031903.1** | **human** | 0 | 0 | 0 | 0 | 0 | 0 | 0 | 0 | 0 | 0 | 3 | 100 | **9** |
| **275mcc** | **pig** | 2 | 100 | 0 | 0 | 0 | 0 | 0 | 0 | 0 | 0 | 0 | 0 | **2** |
| **CP057998.1** | **sheep** | 1 | 50 | 0 | 0 | 0 | 0 | 1 | 50 | 0 | 0 | 0 | 0 | **13** |
| **CP074613.1** | **NA** | 0 | 0 | 0 | 0 | 0 | 0 | 0 | 0 | 0 | 0 | 2 | 100 | **10** |
| **NZ_MT230257.1** | **water** | 1 | 50 | 0 | 0 | 0 | 0 | 0 | 0 | 0 | 0 | 1 | 50 | **2** |
| **PE13-096** | **pig** | 0 | 0 | 0 | 0 | 0 | 0 | 0 | 0 | 0 | 0 | 2 | 100 | **1** |
| **3896** | **poultry** | 1 | 100 | 0 | 0 | 0 | 0 | 0 | 0 | 0 | 0 | 0 | 0 | **2** |
| **CP057403.1** | **pig** | 0 | 0 | 0 | 0 | 0 | 0 | 0 | 0 | 0 | 0 | 1 | 100 | **14** |
| **NZ_CP023365.1** | **canine** | 0 | 0 | 0 | 0 | 0 | 0 | 0 | 0 | 0 | 0 | 1 | 100 | **5** |
| **NZ_CP027395.1** | **human** | 0 | 0 | 0 | 0 | 0 | 0 | 0 | 0 | 0 | 0 | 1 | 100 | **9** |
| **NZ_CP042886.1** | **water** | 0 | 0 | 0 | 0 | 0 | 0 | 0 | 0 | 1 | 100 | 0 | 0 | **11** |
| **NZ_CP055601.1** | **pig** | 0 | 0 | 0 | 0 | 0 | 0 | 1 | 100 | 0 | 0 | 0 | 0 | **12** |
| **RHB12-C14-3** | **cattle** | 0 | 0 | 0 | 0 | 0 | 0 | 0 | 0 | 0 | 0 | 1 | 100 | **13** |
